# Higher consumption of ultra-processed foods is associated with increased risk of fracture among adults: findings from China Health and Nutrition Survey

**DOI:** 10.1007/s00394-026-03937-5

**Published:** 2026-03-03

**Authors:** Ming Li, Yohannes Adama Melaku, Zumin Shi

**Affiliations:** 1 Faculty of Medicine, University of Queensland, Level 3, School of Public Health Building, 288 Herston Road, Herston, Brisbane, QLD 4006 Australia; 2 Flinders Health and Medical Research Institute, Flinders University, Adelaide, Australia; 3 Department of Nutrition Sciences, College of Health Sciences, QU Health, Qatar University, Doha, Qatar

**Keywords:** Ultra-processed food, Long-term consumption, Fracture, Adults, China

## Abstract

**Background:**

Although the consumption of ultra-processed foods (UPFs) has been shown to increase the risk of many diet-related chronic diseases, its potential association with fracture risk remains unexplored in Chinese adults.

**Methods:**

This study used data from a cohort of 13,194 adults who participated at least twice of the China Nutrition and Health Survey (CNHS) between 1997 and 2011 (six waves in total), during which information on fractures was collected. Dietary intake was assessed at each survey using a 3-day 24-hour dietary recall method. UPF consumption was calculated as the total intake of foods classified as NOVA group 4. Fracture was self-reported at each survey. Multilevel mixed-effects logistic regression models, accounting for repeated measures, were used to assess the association, adjusting for covariates including sociodemographic, socioeconomic, health, behavioural, and dietary factors.

**Results:**

Mean per capita UPF consumption increased from 11.3 g in 1997 to 41.5 g in 2011. The total number of participants reported having fracture was 1,471 with 1,057 reported incident fractures during the follow-up. The prevalence of fracture was 2.6% in 1997 and 5.3% in 2011. The adjusted odds ratios (95% CI) for fracture for those with mean UPF consumption of 1–49 g/day, 50–99 g/day, and ≥ 100 g/day were 1.45 (1.25–1.69), 1.62 (1.28–2.04), and 1.66 (1.22–2.24), respectively, compared with non-consumers (p for trend < 0.001). There was significant interaction between UPF and residence with the positive association being significant in rural areas but not in urban areas.

**Conclusion:**

Higher UPF consumption was associated with increased risk of fractures, especially in rural areas. Dietary guidelines should integrate this evidence to address the evolving food environment in China and its potential impact on musculoskeletal health.

**Supplementary Information:**

The online version contains supplementary material available at 10.1007/s00394-026-03937-5.

## Introduction

China has undergone dramatic socioeconomic, demographic, and environmental changes over the past three decades, leading to substantial lifestyle changes and a transition in disease burden from infectious disease to non-communicable disease. The Global Burden of Disease (GBD) has reported that musculoskeletal disorders ranked as the leading cause of years lived with disability (YLD) in 2017 worldwide, and have increased by 34.8% in the last 27 years [1]. The most recent GBD data found that musculoskeletal disorders were among the leading burden of non-communicable diseases [2]. From 1990 to 2019, the incidence, prevalence, and YLDs of fractures rose by 70%, 139%, and 122%, respectively, with falls being the primary cause [3]. In China, a national population-based study utilizing data from 97% of tertiary public hospitals revealed that the healthcare system is heavily burdened by fracture care and faces significant financial pressure. For instance, the fracture admission rate was 1.44 per 1,000, with an in-hospital mortality rate of 1.21 per 1,000. The median hospital stay for fracture patients was 10 days, with an average cost of $3,056 per patient [4]. The high burden and associated cost of fractures have become a significant public health issue, requiring timely mitigation as China’s population ages [5].

Diet plays a pivotal role in health, and research in this area has evolved from examining single nutrients and specific food items to investigating broader dietary patterns and food groups [6, 7] along with the environment and societal changes. Among these changes, urbanization and industrialization have had a major impact on the food supply, leading to greater dominance of heavily processed, ready-to-eat products. Today, approximately 75% of all food sold globally is processed [8]. Dietary patterns have also shifted from a traditional diet rich in vegetables to a modern diet characterized by the high consumption of animal-sourced foods, refined grains, and highly processed, sugar-rich, and high-fat foods [9].

According to the NOVA classification, ultra-processed food (UPF) in group 4 are industrial formulations made primarily from food-derived substances with little or no intact whole foods [10, 11]. Some common UPFs include soft drinks, alcoholic drinks, cookies, and reconstituted meat products, flavored yoghurts, breakfast cereals, and packaged breads. Increased body of evidence from national food intake surveys, large cohorts, and interventional studies have shown that increased UPF consumption increases the risk of multiple diet-related chronic diseases including diabetes, cardiovascular diseases, and cancer [12]. In China, studies have indicated that the mean per capita ultra-processed food (UPF) consumption among Chinese adults tripled from 12.0 g in 1997 to 41.5 g in 2011, with the proportion of UPF in the daily diet increasing from 1.0% to 3.6% [13]. Additionally, our analysis of data from a large population-based study revealed that higher UPF intake is associated with increased odds of overweight/obesity in both children and adults [13, 14], diabetes [15], and hypertension [16] which are confirmed by other global studies [12, 17, 18]. However, evidence on the association between UPF consumption and bone health outcomes is limited [19]. Two cross-sectional studies using data from National Health and Nutrition Examination Survey (NHANES) and one prospective cohort study from UK Biobank linked higher UPF to higher risk of osteoporosis among adults by 10–80% [20–22]. No further evidence is available in China, except for a report suggesting that a modern dietary pattern high in animal-sourced food increases fracture incident in adults [23].

Given the growing UPF consumption and the rising fracture burden in China, it is essential to examine their potential relationship. Furthermore, understanding the interplay between dietary patterns, other behavioral factors, and health risks in relation to UPF consumption may provide critical insights for public health strategies. Therefore, the purpose of this study is to estimate the association between UPF consumption and fracture risk, and to investigate the interaction of socio-demographic, other behavioral factors and health conditions with UPF consumption in predicting fracture risk among Chinese adults.

## Method

### Study population

**Fig. 1:**
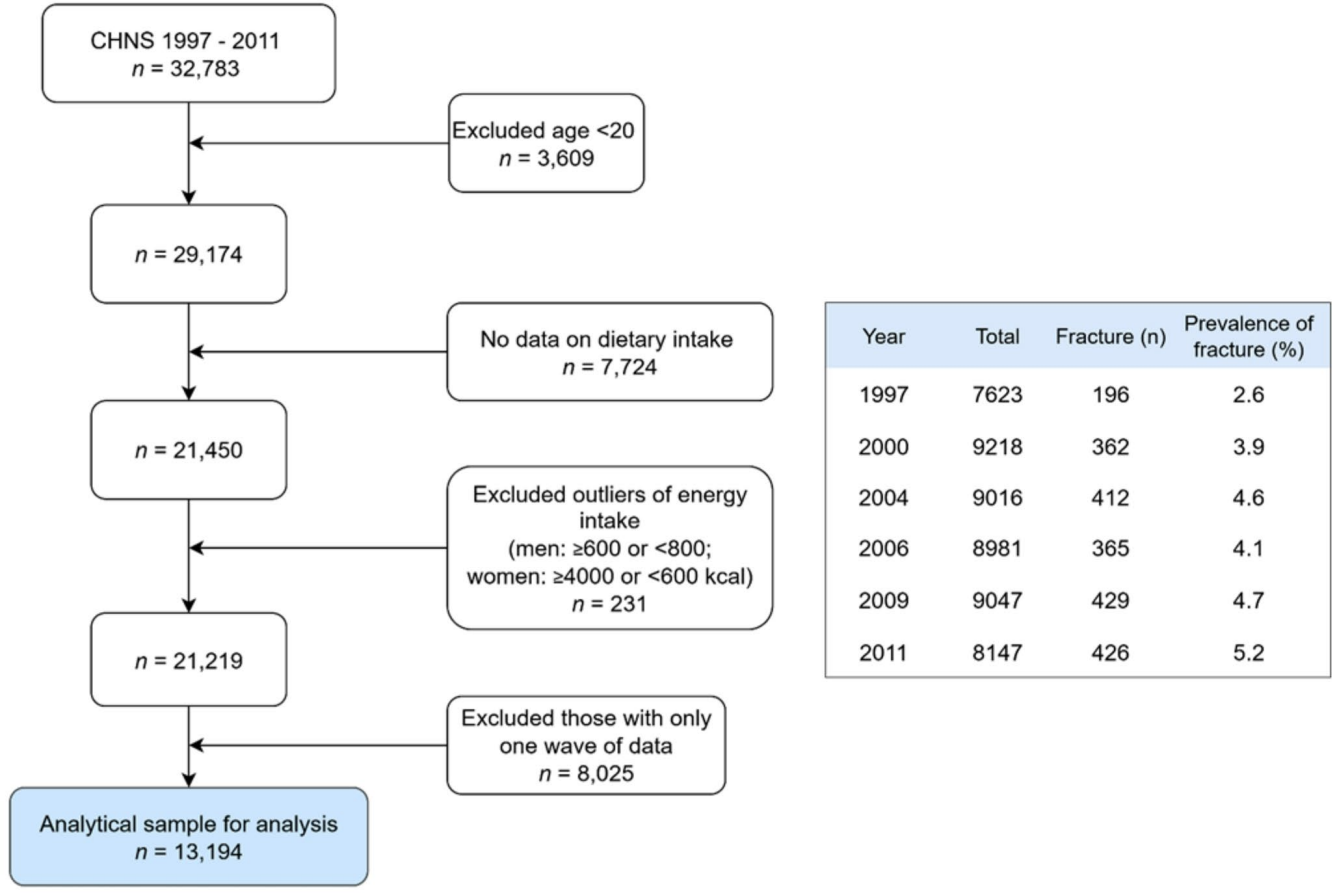
Sample flowchart and prevalence of fracture at each survey wave

We used data from China Health and Nutrition Survey (CHNS). The CHNS is an ongoing, household-based, open cohort study conducted in nine provinces in China [24]. Participants are selected using a multistage random-cluster sampling process in both urban and rural areas. The study has completed eleven waves of data collection (1989, 1991, 1993, 1997, 2000, 2004, 2006, 2009, 2011, 2015 and 2018). All members in the selected households were invited to join and free to stay or leave the subsequent surveys. Over 60% of participants completed all survey rounds during the study period 1989–2006 and more than 80% competed at least two rounds within the same timeframe [24]. This study included data from six waves (1997 to 2011) in consideration of the following: 1). The UPF intake was rare and lacked variation prior to 1997 surveys; 2). The information about fractures was collected since 1997 [23]; and 3). Currently, the 2015 and 2018 dietary survey data are not publicly available. Ultimately, a cohort of 13,194 participants meeting the following inclusion criteria were included (Fig. 1): aged 20 years and above; having attended at least two nutrition surveys between 1997 and 2011; having dietary and fracture information; having plausible energy intake (800–6000 kcal/day for men, and 600–4000 kcal/day for women).

The study was conducted in accordance with the guidelines of the Declaration of Helsinki. All procedures involving research participants were approved by the institutional review boards of the University of North Carolina (USA) and the National Institute of Nutrition and Food Safety (China). Written informed consent was obtained from all participants and/or their guardians.

## Study outcome: fracture

Fracture was self-reported in each wave by the study participants for a question “Have you ever had fracture?” along with age when the first fracture occurred. To determine the calendar year of fracture, first we calculated the difference between the current age (at the interview) and age at first fracture. Then, we subtracted the age differences (in years) from the respective calendar years or waves (when the interview was conducted). In a large cohort study, a self-reported assessment of lifetime fractures, along with age at fractures, was found to be a feasible method to establish incident cases [23]. Therefore, the study outcomes included past fractures, or incident fractures, or both. Specifically, past fractures were defined as having fractures at entry; an incident fracture was defined as having a fracture in the subsequent surveys while not having any fractures at entry, recurrent fracture was defined having at least two fractures based on the response to question “How many times have you had fracture?”.

### Exposure variable: UPF consumption

At each survey, individual dietary intake data were collected by a trained investigator using a 24-hr dietary recall over three consecutive days during home visits [24]. In addition, all foods consumed during the survey period were weighed and assigned to each household member. Specifically, foods and condiments in the home inventory, foods purchased from markets or picked from gardens, and food waste were weighed and recorded by interviewers at the beginning and end of the three-day survey period. The types and amount of food, the type of meal and the place of consumption for a participant were from both dietary recall and the records kept by the individual. Cooking oil and condiments consumption for everyone in the household was estimated using individual energy-weighted intake. Detailed description of the dietary measurement has been published previously [25]. The food data were recorded and converted to nutrient intake using updated food composition Tables [26, 27, 28]. Since 1997, around 3,000 different food items have been categorized into four groups based on the NOVA classification [10]. Any uncertain food items were discussed, and consensus was reached on their processing status. For example, fruity or milky drinks containing sweeteners, preservatives, and other additives were classified as UPF. UPF consumption at each survey for each participant was the sum in grams of all the food items classified as the NOVA group 4 and categorized into four levels: non-consumers, 1–49 g/day (g/d), 50–99 g/d, ≥ 100 g/d. These cut-offs were chosen based on the serving size in the context of Chinese food, where a serving is approximately 50 g (Liang).

### Covariates

Sociodemographic factors were collected at each survey using a structured questionnaire. Socio-economic status: education (low: illiterate/primary school; medium: junior middle school; high: high middle school or higher), per capita annual family income (recoded into tertiles as low, medium and high), urbanization levels (recoded into tertiles as low, medium and high).

Dietary patterns (traditional and modern) were identified using method previously applied in adult population. The traditional pattern was characterized by high intakes of rice, meat, and vegetables, while the modern pattern was highly correlated with fast food, milk, and deep-fried food [8].

Lifestyle factors including smoking, alcohol drinking, sleep, and physical activity were collected from the questionnaires at each survey. Smoking status was categorized as “non-smokers, ex-smokers, and current smokers”. Alcohol consumption was recorded as “yes or no” and the amount of alcohol drinking as a component of UPF was recorded from dietary recall. Sleep duration was recorded as “6, 7–9, and 10 h per day” using data collected since 2004. Physical activity level (metabolic equivalent of task MET) was estimated based on self-reported activities (including occupational, domestic, transportation, and leisure time physical activity) and duration using a compendium of physical activities.

Height was measured without shoes to the nearest 0.2 cm using a portable stadiometer. Weight was measured without shoes and in light clothing to the nearest 0.1 kg on a calibrated beam scale. Body mass index (BMI) was calculated from weight and height. Overweight/obesity was defined as BMI 25 kg/m^2^. Diabetes was self-reported at each survey during 1997–2011 and ascertained as “yes” to either of the following questions: “Has the doctor ever told you that you suffer from diabetes?”; “Did you use any of the treatment methods for diabetes (for example, on diet, weight control, oral medicine, Injection of insulin, Chinese, home remedies, Qigong)?” Hypertension was defined as systolic blood pressure ≥ 140 mmHg and/or diastolic blood pressure ≥ 90 mmHg or having known hypertension.

### Statistical analysis

Baseline characteristics were presented and compared by baseline UPF intake levels of non-consumers, 1–49 g/day (g/d), 50–99 g/d, ≥ 100 g/d. using ANOVA for continuous measures or Chi-square tests for categorical ones. Baseline was the first entry in the Survey.

The association between UPF consumption and fracture was assessed with multilevel mixed effect logistic regression analysis. In the mixed effect logistic model, the study outcome was the history of fracture at each survey. The analytic approach provides flexibility in modelling individual trajectories by allowing random slopes, which let each participant have their own rate of change over time, thereby capturing heterogeneous disease progression and individual variation in how exposures influence outcomes [29]. Unadjusted and adjusted odds ratios (OR) and 95% confidence interval (95% CI) of the fixed part of the mixed effect models were reported, accounting for within person variation that related to the repeated measurements as the random part (for example: age, socio-economic status, lifestyle, UPF consumption). Several models were used: an unadjusted model; Model 1 adjusted for age, sex, and energy intake by using the following STATA command: melogit fracture i.upf_levels age i.sex energy_intake ||id:,or; Model 2 further adjusted for socio-economic status (income, urbanization, and education), fat intake, smoking, and physical activity. Dietary calcium and phosphorus, and health risks such as diabetes, and hypertension were also adjusted additionally. Sensitivity analysis was conducted by including participants attending all 6 rounds of survey in Model 2 (*n* = 3,048). Additional logistic analyses using recurrent fracture as outcome (*n* = 231) were performed to assess the association with cumulative UPF consumption levels and adjusted for the covariates. Cumulative UPF intake for each participant was calculated from all the proceeding survey waves to reduce within-individual variation and to represent long-term habitual intake [14].

The multiplicative interaction between UPF intake and sociodemographic factors (age, sex, residence), lifestyle behaviors, and hypertension on fracture was assessed by introducing a product term in the regression model. All analyses were performed using STATA 18.0 (Stata Corporation, College Station). Statistical significance was set at *P* < 0.05 (two-sided).

## Results

### Population characteristics and UPF consumption

Of the 13,194 participants included in this study, 6,255 were entered in 1997, 1,782 in 2000, 1,125 in 2004, 494 in 2006, and 759 in 2009. The number of participants who took part in 2, 3, 4, 5 and all 6 waves of survey waves were 3,542, 2,093, 2,175, 2,335, and 3,049. At baseline, the mean age of this sample was 43 years (SD 14.9), 47% were males, 31% resided in urbanized area, 30% were smokers, and 32% were alcohol drinkers. The prevalence of overweight/obesity was 21%. The mean daily energy, fat, protein, and carbohydrate intake were 2245 kcal, 65 g, 68 g, and 346 g, respectively.

At baseline, 11,451 participants (73%) reported no UPF intake, while 731 (6%) reported consuming ≥ 100 g/day. Compared with non-consumers, those having UPF ≥ 100 g/day were significantly more likely to be older, males, to have higher education and income, reside in urbanized areas, smoke, drink alcohol and sleep < 6 h per night. They also have higher intake of energy, fat, protein, calcium and phosphorus but lower carbohydrates, consumed more fruit but fewer vegetables; they were more likely to have entered the survey in recent waves, and had a higher prevalence of overweight/obesity, and hypertension. There was no significant difference in the baseline prevalence of diabetes across categories of UPF intake. (Table 1).

**Table 1:** Baseline sample characteristics by levels of UPF intake among adults attending CHNS (*N* = 13,194)

	UPF intake (g/day)					
	None	1–49	50–99	>=100	Total	*p* value
N (%)	10,415 (78.9)	1,352 (10.2)	696 (5.3)	731 (5.5)	13,194 (100.0)	
Year of entry (%)						
1997	6,255 (60.1)	780 (57.7)	301 (43.2)	287 (39.3)	7,623 (57.8)	< 0.001
2000	1,782 (17.1)	197 (14.6)	128 (18.4)	113 (15.5)	2,220 (16.8)	
2004	1,125 (10.8)	144 (10.7)	82 (11.8)	69 (9.4)	1,420 (10.8)	
2006	494 (4.7)	88 (6.5)	55 (7.9)	77 (10.5)	714 (5.4)	
2009	759 (7.3)	143 (10.6)	130 (18.7)	185 (25.3)	1,217 (9.2)	
Age (years) (SD)	43.1 (14.9)	43.2 (15.5)	43.4 (15.1)	45.1 (14.6)	43.2 (15.0)	0.006
Sex (%)						
Men	4,893 (47.0)	650 (48.1)	400 (57.5)	540 (73.9)	6,483 (49.1)	< 0.001
Women	5,522 (53.0)	702 (51.9)	296 (42.5)	191 (26.1)	6,711 (50.9)	
Income (%)						
Low	3,228 (31.4)	306 (22.8)	143 (20.8)	163 (22.6)	3,840 (29.5)	< 0.001
Medium	3,439 (33.4)	437 (32.6)	237 (34.5)	208 (28.8)	4,321 (33.1)	
High	3,620 (35.2)	598 (44.6)	307 (44.7)	350 (48.5)	4,875 (37.4)	
Residence (%)						
Rural	7,147 (68.6)	720 (53.3)	363 (52.2)	424 (58.0)	8,654 (65.6)	< 0.001
Urban	3,268 (31.4)	632 (46.7)	333 (47.8)	307 (42.0)	4,540 (34.4)	
Education (%)						
Low	4,456 (47.7)	447 (36.4)	207 (33.2)	228 (34.1)	5,338 (45.0)	< 0.001
Medium	2,987 (32.0)	401 (32.7)	185 (29.7)	213 (31.9)	3,786 (31.9)	
High	1,904 (20.4)	380 (30.9)	231 (37.1)	227 (34.0)	2,742 (23.1)	
BMI (kg/m^2^)	22.6 (3.2)	22.9 (3.3)	23.0 (3.4)	22.9 (3.1)	22.7 (3.2)	< 0.001
Overweight (%)	1,997 (21.4)	321 (25.5)	173 (26.5)	171 (25.0)	2,662 (22.3)	< 0.001
Self-reported diabetes (%)	166 (1.7%)	14 (1.1%)	10 (1.5%)	12 (1.7%)	202 (1.6%)	0.462
Hypertension (%)	1,421 (15.1%)	223 (17.5%)	116 (17.6%)	145 (21.0%)	1,905 (15.8%)	< 0.001
Fracture (%)	292 (2.9)	61 (4.7)	25 (3.6)	36 (5.0)	414 (3.2)	< 0.001
UPF intake (g/day) (SD)	0.0 (0.0)	22.9 (11.8)	65.3 (13.6)	183.6 (105.6)	16.0 (50.3)	< 0.001
Energy intake (kcal/d) (SD)	2245.3 (635.8)	2194.0 (601.1)	2366.8 (639.9)	2588.3 (742.3)	2265.4 (644.4)	< 0.001
Fat intake (g/d) (SD)	65.3 (35.8)	70.3 (35.3)	79.7 (38.9)	84.5 (41.5)	67.6 (36.7)	< 0.001
Protein intake (g/d) (SD)	67.5 (22.1)	69.7 (22.4)	74.9 (23.9)	78.4 (26.4)	68.7 (22.7)	< 0.001
Carbohydrate intake (g/d) (SD)	346.4 (120.9)	317.7 (114.2)	324.5 (110.6)	321.2 (116.1)	340.9 (120.0)	< 0.001
Calcium intake (mg/d) (SD)	375.9 (341.3)	382.9 (226.5)	416.3 (236.4)	458.4 (283.2)	383.3 (324.1)	< 0.001
Phosphorus intake (mg/d) (SD)	1001.9 (383.2)	977.1 (321.9)	1061.8 (366.3)	1098.7 (376.0)	1007.9 (377.0)	< 0.001
Modern pattern score (SD)	− 0.0 (1.0)	0.1 (0.9)	0.1 (1.0)	0.2 (1.0)	0.0 (1.0)	< 0.001
Traditional dietary pattern score (SD)	− 0.3 (0.7)	0.1 (0.9)	0.4 (1.0)	0.9 (1.3)	− 0.2 (0.9)	< 0.001
Fruit intake (g/d) (SD)	17.0 (70.6)	36.1 (94.1)	45.7 (97.7)	60.9 (118.0)	22.9 (79.2)	< 0.001
Fresh vegetable intake (g/d) (SD)	283.5 (175.8)	265.1 (167.4)	261.7 (167.5)	267.1 (169.3)	279.5 (174.3)	< 0.001
Physical activity (MET) (SD)	139.6 (116.9)	131.1 (113.2)	138.7 (122.1)	144.3 (117.4)	138.9 (116.8)	0.055
Alcohol drinking (%)						
No	6,805 (67.9)	814 (62.1)	338 (49.4)	248 (34.4)	8,205 (64.4)	< 0.001
Yes	3,212 (32.1)	497 (37.9)	346 (50.6)	473 (65.6)	4,528 (35.6)	
Smoking (%)						
Non-smoker	7,046 (69.0)	892 (67.2)	415 (59.9)	349 (48.1)	8,702 (67.2)	< 0.001
Ex-smokers	127 (1.2)	21 (1.6)	21 (3.0)	21 (2.9)	190 (1.5)	
Current smokers	3,032 (29.7)	414 (31.2)	257 (37.1)	356 (49.0)	4,059 (31.3)	
Sleep duration * (hours) (%)						
6–9	1,858 (80.7)	297 (80.1)	199 (74.8)	260 (79.8)	2,614 (80.0)	0.308
<=6	180 (7.8)	29 (7.8)	24 (9.0)	31 (9.5)	264 (8.1)	
>9	265 (11.5)	45 (12.1)	43 (16.2)	35 (10.7)	388 (11.9)	

Dietary patterns from factor analysis: traditional pattern characterized by high intake of rice, pork, and vegetables, and low intake of wheat; a modern dietary pattern had high intake of fruit, soy milk, egg, milk, and deep-fried products [9]

Calcium intake and phosphorus intake obtained from China food composition Tables (26–28).

*Sleep duration self-reported at the 2004, 2006, 2009 and 2011 surveys.

### UPF consumption and fracture risk

At entry, 414 participants reported a history of fracture, with half experiencing the event at the age of ≥ 35 years and 25% at the age ≥ 50 years. The baseline prevalence of fracture was 2.6% in 1997 increased to 5.2% in 2011 (Fig. 1). During the study period, 1,057 participants experienced incident fractures, and 1.8% (*n* = 231) reported recurrent fractures in the last survey. The baseline prevalence of fractures for adults with daily UPF consumption of none, 1–49 g, 50–99 g, and ≥ 100 g was 2.9%, 4.7%, 3.6%, and 5.0%, respectively.

Table 2 presents the odds ratios (OR) and 95% confidence intervals (CI) for the association between UPF intake and fracture risk among adults in the CHNS (1997–2011) from the fixed part of the multilevel regression analyses. In the unadjusted model, higher UPF intake was associated with a significantly increased fracture risk, with ORs of 1.81 (95% CI: 1.58–2.08) for 1–49 g/day, 2.14 (95% CI: 1.72–2.65) for 50–99 g/day, and 2.15 (95% CI: 1.62–2.85) for ≥ 100 g/day (p-trend < 0.001). After adjusting for age, sex, intake of energy and fat, income, education, residence (urban/rural), smoking and physical activity, the association was attenuated but significant and the corresponding ORs (95% CI) were 1.45 (1.25–1.69), 1.62 (1.28–2.04), and 1.66 (1.22–2.24), across increasing UPF intake categories (p-trend < 0.001). Further adjustments for diabetes and/or hypertension, dietary calcium or phosphorus intake, or dietary pattern did not change the associations. Sensitivity analysis yielded similar results when 3,048 participants attending all 6 surveys were included.

**Table 2:** Odds ratio (95%CI) for fracture by UPF intake levels among adults attending CHNS 1997–2011

	UPF intake (g/day)				
	None	1–49	50–99	>=100	*p* for trend
Unadjusted	1.00	1.81 (1.58–2.08)	2.14 (1.72–2.65)	2.15 (1.62–2.85)	< 0.001
Model 1	1.00	1.51 (1.31–1.73)	1.77 (1.43–2.20)	1.83 (1.37–2.43)	< 0.001
Model 2	1.00	1.45 (1.25–1.69)	1.62 (1.28–2.04)	1.66 (1.22–2.24)	< 0.001
Model 2 + diabetes	1.00	1.47 (1.26–1.71)	1.61 (1.27–2.04)	1.67 (1.23–2.27)	< 0.001
Model 2 + diabetes + hypertension	1.00	1.47 (1.26–1.72)	1.65 (1.30–2.09)	1.64 (1.20–2.25)	< 0.001
Model 2 + calcium intake	1.00	1.44 (1.24–1.68)	1.60 (1.27–2.03)	1.64 (1.21–2.22)	< 0.001
Model 2 + phosphorus intake	1.00	1.45 (1.25–1.69)	1.62 (1.28–2.04)	1.67 (1.23–2.26)	< 0.001
Model 2 + dietary pattern	1.00	1.38 (1.18–1.61)	1.48 (1.16–1.88)	1.43 (1.04–1.96)	< 0.001
Sensitivity analysis	1.00	1.68 (1.32–2.15)	1.72 (1.11–2.66)	1.91 (1.06–3.44)	< 0.001

OR (95% CI) from multilevel mixed effect logistic regression analysis

P for trend from models using UPF intake levels as a continuous variable

Model 1 adjusted for age, sex, and energy intake

Model 2 further adjusted for intake of fat, income, education, residence (urban/rural), smoking, and physical activity

Dietary patterns from factor analysis: traditional pattern characterized by high intake of rice, pork, and vegetables, and low intake of wheat; a modern dietary pattern had high intake of fruit, soy milk, egg, milk, and deep-fried products [9]

Calcium intake and phosphorus intake obtained from China food composition Tables (26–28).

Sensitivity analysis: including participants attending all 6 rounds of surveys in Model 2 (n = 3,048)

All the adjusted variables (except sex) are treated as time-varying covariates.

Using recurrent fracture reported in the last survey as outcome (*n* = 231), the prevalence for adults with daily UPF consumption of none, 1–49 g, 50–99 g, and ≥ 100 g was 1.3%, 1.9%, 2.5%, and 2.4%, respectively with the corresponding unadjusted OR (95% CI) of 1.00, 1.46 (1.09–1.98), 1.90 (1.26–2.88), and 1.89 (1.12–3.19) (p for trend < 0.001). Multivariable logistic regression showed that the adjusted odds ratios (95% CI) were 1.20 (0.85–1.69) for 1–49 g/day, 1.51 (0.93–2.43) for 50–99 g/day, and 1.92 (1.10–3.37) for ≥ 100 g/day of UPF intake (p-trend < 0.001). The association did not change substantially when further adjusted for dietary pattern, calcium or phosphorus, and health (**Supplement** Table 1).

The association between UPF consumption and fractures differed by residential area. Specifically, participants living in rural areas who consumed ≥ 100 g/day of UPFs had higher odds of fractures (OR 2.15, 95% CI 1.49–3.11), whereas those living in urban areas with the same level of UPF intake did not (OR 1.04, 95% CI 0.61–1.77; p for interaction = 0.029). The odds of fracture increased with age, particularly among those aged over 40 years while those aged 40–50 years had significantly higher risk of fracture associated with UPF consumption of 1–49 g/day (OR 1.59; 95% CI: 1.27–1.99), 50–99 g/day (OR 2.25 ; 95% CI: 1.62–3.13), and ≥ 100 g/day (OR 1.75; 95% CI: 1.14–2.69) (P for trend < 0.001). The increased odds of fracture associated with UPF consumption was consistent across subgroups by sex, smoking and drinking status and hypertension status (Table 3).

**Table 3:** Subgroup analyses of the association (OR (95% CI)) between UPF intake and fracture

	UPF intake (g/day)					
	None	1–49	50–99	>=100	*p* for trend	*p* for interaction
Age						0.111
20–39	1.00	1.38 (0.96–1.97)	1.18 (0.67–2.08)	1.62 (0.81–3.22)	0.092	
40–50	1.00	1.59 (1.27–1.99)	2.25 (1.62–3.13)	1.75 (1.14–2.69)	< 0.001	
50+	1.00	1.26 (0.97–1.65)	1.30 (0.84-2.00)	1.64 (0.93–2.90)	0.035	
Sex						0.192
Men	1.00	1.35 (1.09–1.67)	1.59 (1.18–2.13)	1.77 (1.25–2.50)	< 0.001	
Women	1.00	1.54 (1.24–1.91)	1.64 (1.11–2.42)	1.28 (0.66–2.47)	< 0.001	
Residence						0.029
Rural	1.00	1.52 (1.26–1.83)	1.89 (1.41–2.54)	2.15 (1.49–3.11)	< 0.001	
Urban	1.00	1.31 (1.00-1.70)	1.23 (0.84–1.82)	1.04 (0.61–1.77)	0.326	
Smoking						0.573
Non-smoker	1.00	1.55 (1.28–1.86)	1.89 (1.39–2.58)	1.78 (1.14–2.80)	< 0.001	
Ex-smokers	1.00	1.64 (0.76–3.50)	1.47 (0.51–4.27)	1.30 (0.37–4.63)	0.447	
Current smokers	1.00	1.21 (0.92–1.58)	1.39 (0.96–2.02)	1.57 (1.02–2.42)	0.016	
Alcohol drinking						0.151
No	1.00	1.48 (1.23–1.79)	1.75 (1.25–2.43)	1.00 (0.56–1.80)	< 0.001	
Yes	1.00	1.33 (1.05–1.68)	1.41 (1.03–1.93)	1.82 (1.28–2.58)	< 0.001	
Hypertension						0.362
No	1.00	1.46 (1.23–1.75)	1.82 (1.39–2.39)	1.49 (1.04–2.15)	< 0.001	
Yes	1.00	1.43 (1.06–1.94)	1.54 (0.96–2.48)	2.02 (1.15–3.53)	0.004	

OR (95% CI) from multilevel mixed effect logistic regression analysis

p for trend from models using UPF intake levels as a continuous variable

p for interaction from models including an interaction term of UPF and the corresponding variable

Models adjusted for age, sex, intake of fat and energy, household income, education, residence (urban/rural), smoking, and physical activity

All the adjusted variables (except sex) are treated as time-varying covariates.

Supplementary Table 1 Odds ratio (95% CI) for recurrent fractures by accumulative UPF intake levels among adults attending CHNS 1997–2011

Among the socio-demographic, behavioral, and health-related factors examined in this study, age was significantly related to fracture. Each additional year of age was associated with 4% increased odds of fracture (aOR 1.04, 95% CI 1.02–1.05), with the highest risk observed among participants aged ≥ 40 years who consumed ≥ 100 g/day of UPF (aOR 1.81, 95% CI 1.18–2.77) (*P* < 0.01).

## Discussion

The study demonstrated a dose-responsive association between increased UPF consumption and higher odds of fracture among Chinese adults (aged ≥ 20 years) attending CHNS during 1997–2011. Specifically, adults consuming 1–49 g/day, 50–99 g/day, and ≥ 100 g/day of UPF had 46%, 64%, and 69% higher odds of fracture, compared to non-consumers, respectively. The association was independent of socio-demographic, dietary calcium and/or phosphorus, dietary pattern, and behavioral factors, and health risk such as overweight/obesity, diabetes, and hypertension. In addition, the effect size varied by residential location, with adults in rural areas experiencing more than twice the odds of fracture at UPF intake ≥ 100 g/day, while no significant association was observed among their urban counterparts.

Half of the self-reported fracture occurred in adults aged ≥ 35 years, and over a quarter occurred among those aged ≥ 50 years among the Chinese adults. These findings highlight the need to develop effective strategies to prevent fractures in older adults in facing with China’s rapidly aging population [3, 5]. To start, evidence of the burden of fracture, lifestyle, health and environmental factors is required to identify the high-risk population. The China National Fracture Study, a nationally representative population-based study of more than half a million people, reported the highest incidence in women aged 55–64 years of 7.04 (6.06–8.01) per 1000 people in 2014, with over half (58%) being fragility fractures caused by low-energy trauma (slips, trips, and falls from standing height) [30]. Further, the China Osteoporosis Prevalence Study, which included 20,416 nationally representative participants in 2017–2018, reported that among adults aged > 40 years, the prevalence of osteoporosis was 5.0% in men and 20.6% in women, assessed using x-ray absorptiometry image, and the prevalence of clinical fracture in the past 5 years was approximately 4% [31]. These estimates are broadly consistent with our estimate of 3.2% among adults aged over 18 years. Fracture history, low socioeconomic status, alcohol consumption, and average sleep time less than 7 h were independent risk factors for traumatic fracture in adults in addition to older age [30].

Adding to the other studies, we found higher consumption of UPF associated with increased likelihood of fracture in a dose-responsive manner. Although no prior studies have examined this relationship among Chinese population, cross-sectional evidence from US, Korea consistently indicated similar positive associations with fracture and/or bone health [20, 22, 32]. For example, higher UPF intake was linked to 52% increased odds of osteoporosis [22], and a 1.9% increase in self-reported fracture for each 1% increase in UPF consumption [22]. In addition, higher UPF intake was associated with 58% greater odds of low bone mineral density in Americans [20] and lower femoral neck and total femur bone mass in Koreans [32]. Consistently, a prospective cohort study from UK Biobank data revealed a 9% higher risk of osteoporosis associated with high UPF intake after a median follow up of 13.3 years of 141,577 adults [21]. Although the strength of the association from the current study is not comparable with others due to the differences in study type, the measurement of UPF intake and study outcomes, and statistical analysis, the direction of the associations are universal. A recent scope review summarized broadly the deleterious role of UPF on bone health with majority studies reporting osteoporosis or bone mineral density or joint health among not only in adults but also among children, adolescents and young adults, more high-quality research is needed to fill the knowledge gap [19]. Given the widespread availability, aggressive marketing, and affordability of UPFs have led to their increasing consumption across all age groups, including children, adolescents, and older adults, who are particularly vulnerable to bone fragility and joint diseases, and the potential public health burden of UPF consumption on bone health, the need for dietary interventions and policy changes should be reinforced [33].

This study also revealed that adults residing in rural areas were more likely to have fractures associated with increased UPF consumption comparing to “0” consumption (1.52 for 1–49 g/day, 1.91 for 50–99 g/day, and 2.19 for ≥ 100 g/day), while this trend was not observed in urban adults. This could be due to the insufficient sample of fracture in urban areas, requiring further exploration using other national data. Also, it could suggest affordability and easy access to UPF, more fractures cases of combine traumatic injury and osteopenia fracture in rural areas that could not be differentiated in this study.

### Potential mechanisms

The underly mechanism of UPF consumption on increased risk of fracture has not been fully explicated, calling for more studies to elucidate the causal relationship between UPF consumption and fracture and/or bone health. Several mechanisms may explain the association, including direct bone mineral density reduction, increased systemic inflammation, and potential disruption of mineral metabolism through phosphate additives. A recent animal experiment highlighted the severe impact of consuming UPF on the growing skeleton, showing that young rats fed UPF rich in fat and sugar suffer from growth retardation due to lesions in their tibial growth plates. The bone mineral density decreases significantly, and the structural parameters of the bone deteriorate, presenting a sieve-like appearance in the cortices and poor trabecular parameters in long bones and vertebrae. This results in inferior mechanical performance of the entire bone with a high fracture risk [34]. Another laboratory study also suggests that 6-week regular UPF diet composing of bread roll, hamburger, tomatoes, lettuce, ketchup (excluding onion and pickles) and chips, alters the gut microbiome and has negative outcomes on bone parameters and bone marrow adiposity in rats after birth [35]. On the other hand, the supplementation with multi-vitamins-minerals positive effect has improved bone growth and quality that was followed by damage to the rats’ kidneys with modifications in inflammation and vitamin-D metabolism induced by UPF feeding [36]. Furthermore, this intervention study also demonstrated that a nutritional rescue approach partially improved the structural and mechanical parameters of bone [36]. Inflammatory markers such as C-reactive protein (CRP), neutrophil-to-lymphocyte ratio (NLR), and systemic inflammation index (SII) mediate UPF and osteoporosis pathway, accounting for 2.76–3.30% of this association [21], while epidemiological studies have linked UPF consumption to low-grade inflammation [21, 37] as summarized in a recent review [38]. Emerging studies have suggested that UPFs can negatively impact the gut microbiome, reducing the abundance of beneficial bacteria like *Acetatifactor* that produce short-chain fatty acids, which can play a role in bone health by promoting bone formation and inhibiting bone resorption [39, 40]. Others potential explanations could include deficiencies in essential bone-building nutrients like calcium, phosphorus, and vitamin A, C, and D, which are crucial for bone density and strength, especially during and after menopause due to high UPF consumption. In addition, UPFs can contribute to metabolic dysregulation, including compromised glycemic homeostasis and increased risk of metabolic syndrome, which indirectly impacts bone health. However, our study showed the association was independent of dietary intake of calcium and the metabolic risks such as diabetes, and hypertension, suggesting involvement of multiple mechanisms not solely due to dietary nutrient profile deterioration. For example, excessive phosphate-based food additives in UPFs may disrupt bone and mineral metabolism in humans [41] either directly through tissue/vessel calcification or indirectly through the release of mineral-regulating hormones, parathyroid hormone, and fibroblast growth factor-23 [42]. While some plausible mechanisms have been investigated, more research is needed to establish causal mechanisms.

### Strengths and limitations

A key strength of this study is the use of a large nationwide sample, allowing for robust population-level inferences. The study period (1997–2011) encompassed a critical phase of China’s nutrition transition, capturing shifts in dietary patterns over time. UPF intake was assessed using a combination of 3-day dietary recalls and household food inventory data, providing a comprehensive and reliable estimate of long-term consumption. This methodological approach enhances the accuracy of dietary exposure assessment and strengthens the validity of the observed associations. The application of multilevel mixed effect modelling maximized the inclusion of all repeated measures over decades, allowing to account for long-term diet variations and to control for time-varying confounding in this open cohort study design in the investigation of association. A series of sensitivity analyses confirmed the robustness of the results either by including only those attending all waves of survey, or by using recurrent fracture as an outcome and cumulative UPF consumption as a long-term exposure factor in logistic regression analysis. Potential confounding factors including sociodemographic, behavioral, health, dietary factors, calcium and phosphorus were adjusted.

Limitation should be noted when interpreting the findings from this study. Firstly, fracture was self-reported. We could not differentiate fracture as an external injury or pathological event; there were neither biochemical markers to validate blood calcium, phosphorus, and vitamin D or inflammation levels, nor any bone images to verify bone mineral density or any further diagnosis as to the fracture site, which hampered the understanding of the underlying mechanism behind the increased risk of fracture associated with higher UPF consumption. Further, misclassification of UPF was possible due to incomplete records on food processing methods in the CHNS survey, which was not specifically matched with NOVA classification. The ascertainment of food items might not be subtle in reflecting the complexity of food processing and variabilities in additive composition between brands for a similar type of product, and some food items could only be grouped, therefore, the association between UPF and fracture could be biased. Finally, we cannot rule out the role of residual and other potential confounding factors, such as medication use (e.g., psychoactive medications, glucocorticoid use), supplement use (hormonal and dietary), and other health risks (e.g., kidney diseases, gout), which could potentially overestimate the associations.

## Conclusion

Higher UPF consumption was associated with an increased odds of fracture among Chinese adults. Dietary guidelines should integrate this evidence to address the evolving food environment in China and its potential impact on fracture risk. Further investigations to understand the mechanism through which UPF consumption and fracture risk are warranted.

## Electronic Supplementary Material

Below is the link to the electronic supplementary material.

Supplementary Material 1

## Data Availability

The current research uses data from the China Health and Nutrition Survey (CHNS). Data described in the manuscript, code book, and analytic code are made publicly and freely available without restriction at https://www.cpc.unc.edu/projects/china accessed on 15 January 2025.
