# Supplementary material for: Higher consumption of ultra-processed foods is associated with increased risk of fracture among adults: findings from China Health and Nutrition Survey
Source: Eur J Nutr. 2026 Mar 3;65(2):79. doi: 10.1007/s00394-026-03937-5 (PMC12956972; doi:10.1007/s00394-026-03937-5)
Supplement: Supplementary file 1 — Supplementary Material 1 [file 394_2026_3937_MOESM1_ESM.docx]

Supplementary Table 1 Odds ratio (95% CI) for recurrent fractures by accumulative UPF intake levels among adults attending CHNS 1997-2011

|  | UPF intake (g/day) | | | |  |
| --- | --- | --- | --- | --- | --- |
|  | None | 1-49 | 50-99 | >=100 | p for trend |
| No. of participants in the last survey | 5215 | 5813 | 1426 | 740 |  |
| No. (%) of participants with recurrent fracture | 68 (1.3%) | 110 (1.9%) | 35 (2.5%) | 18 (2.4%) |  |
| Unadjusted | 1.00 | 1.46 (1.08-1.98) | 1.90 (1.26-2.88) | 1.89 (1.12-3.19) | <0.001 |
| Model 1 | 1.00 | 1.39 (1.02-1.89) | 1.90 (1.25-2.88) | 2.03 (1.19-3.48) | <0.001 |
| Model 2 | 1.00 | 1.20 (0.85-1.69) | 1.51 (0.93-2.43) | 1.92 (1.10-3.37) | <0.001 |
| Model 2 + dietary pattern | 1.00 | 1.20 (0.84-1.70) | 1.55 (0.94-2.55) | 1.95 (1.07-3.55) | <0.001 |
| Model 2 + calcium intake | 1.00 | 1.18 (0.83-1.66) | 1.47 (0.91-2.37) | 1.88 (1.07-3.30) | <0.001 |
| Model 2 + phosphorus intake | 1.00 | 1.18 (0.84-1.67) | 1.50 (0.93-2.41) | 1.93 (1.10-3.38) | <0.001 |
| Model 2 + diabetes + hypertension | 1.00 | 1.09 (0.77-1.56) | 1.49 (0.92-2.40) | 1.90 (1.08-3.35) | <0.001 |

*OR (95% CI) from logistic regression analysis using each participant’s last survey data, with recurrent fracture (reported times of fracture ≥2) as the outcome and cumulative mean UPF as the exposure variable. The number of participants with recurrent fractures was 231.*

*p for trend from models using UPF intake levels as a continuous variable*

*Model 1 adjusted for age, sex, and energy intake*

*Model 2 further adjusted for intake of fat, income, education, residence (urban/rural), smoking, and physical activity*

*Dietary patterns from factor analysis: traditional pattern characterized by high intake of rice, pork, and vegetables, and low intake of wheat; a modern dietary pattern had high intake of fruit, soy milk, egg, milk, and deep-fried products (9)*

*Calcium intake and phosphorus intake obtained from China food composition tables (26-28).*
